# Temporal Changes in Splenic Immune Cell Populations following Infection with a Very Virulent plus MDV in Commercial Meat-Type Chickens

**DOI:** 10.3390/v16071092

**Published:** 2024-07-06

**Authors:** Nagwa Khaled, Raveendra R. Kulkarni, Tobias Käser, Isabel M. Gimeno

**Affiliations:** 1 Department of Population Health and Pathobiology, College of Veterinary Medicine, North Carolina State University, Raleigh, NC 27607, USA; nkkhaled@ncsu.edu (N.K.); rrkulkar@ncsu.edu (R.R.K.); tobias.kaeser@vetmeduni.ac.at (T.K.); 2 Department of Virology, Faculty of Veterinary Medicine, University of Sadat City, Monofiya 23511, Egypt

**Keywords:** Marek’s disease virus, immunosuppression, spleen, maternal antibodies, chickens

## Abstract

Marek’s disease virus (MDV) can cause severe immunosuppression in chickens. Our previous study showed that infection with very virulent plus (vv+) MDV strains of one-day-old commercial meat-type chickens possessing maternal antibodies against MDV resulted in severe depletion of splenocytes at 28–30 days of age. In the present study, we have investigated the effect of vv+MDV strain 686 on splenic immunophenotypes at 6, 20, and 30 days post-infection (dpi). Both live and dead cells were analyzed, and the data were statistically compared to the uninfected control. The results revealed a decrease in the total live cell population starting on day 20, primarily affecting B cells, CD8β+, and gamma delta (γδ) T cells, while the frequencies of both live and dead CD3+ and CD4+ T cells were increased. The MHC-I expression of CD3+ and CD4+ T cells was higher at 20 and 30 dpi, while the expression of MHC-II on these cells was downregulated at 6 dpi but was upregulated at 30 dpi. Collectively, these results suggest that maternal antibodies seem to delay the negative effects of vv+MDV on the splenic lymphoid populations, albeit being non-protective. Our results emphasize the importance of MD vaccination in vv+MDV endemic areas.

## 1. Introduction

Marek’s disease (MD) is a lymphoproliferative disease of chickens caused by an oncogenic virus known as Marek’s disease virus, or MDV (Gallid alpha herpesvirus 2 or GaAHV2). Although vaccines have played a crucial role in controlling MD, it remains a significant concern in the poultry industry as the virus has evolved towards more virulence and is capable of inducing tumors and other non-neoplastic syndromes, such as severe immunosuppression, in vaccinated flocks [1].

MDV belongs to the family Orthoherpesviridae, subfamily Alphaherpesvirinae, genus Mardivirus, and specie Mardivirus gallidalpha2. There are two other closely related species in the genus Mardivirus (Mardivirus gallidalpha3 and Mardivirus meleagridalpha1). Viruses from these three species were formerly classified into MDV-1, MDV-2, and herpesvirus of turkey, or HVT, respectively. GaAHV2 (belonging to species Mardivirus gallidalpha2 and formerly known as MDV-1) includes all oncogenic viruses, and it is the causative agent of MD, whereas viruses belonging to the other two species are non-oncogenic and are used as vaccines against MD [2,3]. In this study, the term MDV will refer only to GaAHV2. MDV has increased in virulence over the last 60 years, and it can be further classified into pathotypes based on their ability to overcome vaccine-induced immunity [4]. The following four pathotypes have been described: mMDV (mild), vMDV (virulent), vvMDV (very virulent), and vv+MDV (very virulent plus) [4]. As the virus has increased in virulence, it has acquired the following novel features: mMDV induces only inflammation in peripheral nerves; vMDV also induces neoplastic transformation of lymphocytes; and vvMDV, and even more vv+MDV, have an increasing ability to induce disease in the central nervous system (transient paralysis syndrome) [5]; in the eye (panophtalmitis) [6]; and cause severe immunosuppression [7].

MDV-induced immunosuppression (MDV-IS) is divided into three different phases [7,8,9,10]. The early MDV-IS occurs during early cytolytic infection in the lymphoid organs about 4–6 days post-infection (dpi) in chickens lacking maternal antibodies. The late MDV-IS due to virus reactivation, only caused by vv+MDV, can occur in chickens bearing maternal antibodies even if they are properly protected against tumors by vaccination [11]. The late MDV-IS due to tumors occurs when lymphomas are developing [9]. Maternal antibodies do not protect against the development of tumors nor protect against MDV-IS due to tumors; however, vaccination does. The three phases of MDV-IS are not related to each other and can occur independently [12]. In chickens lacking maternal antibodies, there is a temporal separation between early MDV-IS (4–6 dpi) and the two phases of late MDV-IS (21 dpi onwards). However, in chickens with maternal antibodies, it is possible that the three phases of MDV-IS overlap each other.

Early MDV-IS is caused by the destruction of T and B cells in lymphoid organs, resulting in severe lymphoid organ atrophy in chickens lacking maternal antibodies. Maternal antibodies are able to protect against the severe damage that MDV produces in the lymphoid organs [13,14,15] and effectively reduce the initial productive viral infection and acute inflammatory response observed in lymphoid organs [16]. The precise mechanism through which maternal antibodies mitigate infection remains unidentified. However, it is speculated that they may interfere with the cell-to-cell spread of the virus [17] and neutralize free virus particles during initial entry, thereby reducing the infectious dose. However, protection conferred by maternal antibodies can be compromised by high doses of highly virulent MDV strains [14,16,18,19]. Even though maternal antibodies can mitigate lymphoid organ atrophy induced by highly virulent strains to some extent [20,21], such protection might not be sufficient to avoid immunosuppression [22,23].

Broiler chickens (maternal antibody-positive) typically are slaughtered before they develop gross tumors (5–8 weeks) [22]. Hence, in some countries, broiler chickens are not vaccinated, and if they are, they generally receive the least protective MD vaccine (HVT) [24]. In a previous study, we demonstrated that infection with vv+MDV strains led to severe destruction of splenocytes at 28–30 days of age in broiler chickens [25]. In the current study, using flow cytometry, we have evaluated the temporal (6, 20, and 30 dpi) phenotypic changes that vv+MDV 686 strain causes in the spleen of commercial meat-type chickens bearing maternal antibodies.

## 2. Materials and Methods

### 2.1. Experimental Animals

Commercial-specific pathogen-free SPAFAS chickens (Charles River SPAFAS, N Franklin, CT, USA) were used as MDV-shedder chickens. Female commercial meat-type chickens bearing maternal antibodies (came from dams vaccinated with vaccines of the three serotypes HVT, SB-1, and CVI988) were used as experimental chickens.

### 2.2. Viruses and Vaccine

Serotype 1 MDV strain 686 at 10 passages in duck embryo fibroblasts [26] was used to challenge the SPAFAS shedder chickens. Shedders were vaccinated with a commercial HVT vaccine [27] to avoid mortality due to transient paralysis before they could transmit the infection.

### 2.3. Experimental Design

This study was conducted under the guidance and approval of the North Carolina State University Institutional Animal Care & Use Committee (IACUC). For the MDV challenge, we use shedder chickens (SPAFAS) vaccinated with HVT at 18 days of embryonation via the amniotic route. These chickens were then challenged with 500 PFU of the vv+MDV strain 686 via subcutaneous injection one day post hatching. Contact exposure of the experimental chickens was confirmed by evaluating the viral DNA load of the oncogenic virus in the feather pulp of every chicken sampled by qPCR, as reported [28]. Shedders were kept in isolation in a floor pen for 15 days prior to the beginning of the experiment. Eighty one-day-old meat-type chickens were divided into two groups of 40 chickens each. One of the groups was comingled with the 15-day-old shedder chickens so that they could be infected with MDV at the day of birth. The second group remained unchallenged as negative control. Birds from both groups were euthanized at 6 dpi (21 chickens per group), 20 dpi (7 chickens per group), and 30 dpi (7 chickens per group) to collect spleens for flow cytometry. Five extra chickens were included in each group to account for potential mortality.

### 2.4. Spleen Single-Cell Suspension

Spleen tissues (n = 7; three spleens pooled per sample at 6 days and one spleen per sample at 20 and 30 days) were collected in 10 mL of R10 growth medium (RPMI with 10% FBS, 2.5 mL gentamicin, 5 mL penicillin/streptomycin, and 0.175 mL 2-mercaptoethanol) on ice and then homogenized using a plunger from a sterile 3-cc syringe. With gentle circular motion, the dissociated tissue and cell suspensions were passed twice through 40 µm cell strainers (Fisher scientific^®^, Pittsburgh, PA, USA 15275USA) to collect single cells. After centrifugation at 400× *g* for 10 min at 4 °C, the pellet was loosened by gentle flicking. Moreover, 1× ACK (ammonium-chloride potassium) lysis buffer (Biowhittaker^®^, Lonza, VWR Scientific, Mississauga, ON, Canada) was used to lyse red blood cells. The ACK lysis buffer was added at a volume of 1.5 mL per pellet and left at room temperature for 5 min. Splenocytes were finally resuspended in 5 mL R10 growth medium and counted using a hemocytometer and trypan blue. Cell density was adjusted to a final concentration of 10^7^ cells/mL.

### 2.5. Flow Cytometry-Based Cellular Analysis

Cells were plated on 96-well round-bottom plates, with each well containing 10^6^ cells in 100 μL FACS buffer. Primary antibodies were added to each well (0.5–1 μg/10^6^ cells) and stained for 30 min on ice with fluorescent mouse monoclonal antibodies (Southern Biotech, Birmingham, AL, USA). The cells were stained in five different panels of antibody staining due to the paucity of chicken antibody reagents available in multicolor formats (Supplementary Table S1). Because of scarcity of reagents during the pandemic, on day six, only three antibody panels could be stained. In all the panels, cell viability dye, Live/Dead™ near-infrared (Invitrogen, Carlsbad, CA, USA), was used to exclude dead cells. Then, cells were washed twice in FACS buffer and centrifuged at 1650× *g* for 5 min at 4 °C. After the cells were fixed in 4% paraformaldehyde (PFA) for 10 min, they were centrifuged, resuspended in 200 μL of PBS, transferred to 5 mL round-bottom polystyrene tubes (BD Falcon 352052), kept covered, and placed on ice until analysis. Alongside these samples were also negative controls (two replicates each), including unstained cells and single-color controls consisting of cells treated with one of the monoclonal antibodies mentioned above, so that each antibody was represented individually and served as compensation controls. These wells received 1 μL of the antibody and 100 μL of FACS buffer. Fluorescence minus one gating control was also used. Data acquisition was executed using an LSR-II flow cytometer (BD Biosciences, Franklin Lakes, NJ, USA). Data analysis was performed using Flow Jo software v10 (Tree Starr, Ashland, OR, USA). The gating strategy included the exclusion of doublet cells through forward and side scatter plotting using area (A), height (H), and width (W), followed by gating on live cells and dead cells and further gating of the specific cell population in each panel (Supplementary Figures S1–S5). Frequencies of different cell populations were determined and analyzed using the following statistical method.

### 2.6. Statistical Analysis

All the data were analyzed at all time points using GraphPad Prism version 10 (GraphPad Software, LLC Inc., Boston, MA, USA). The data were first tested for normal distribution (Shapiro–Wilk test), followed by Student’s unpaired *t*-test to compare between the 686-infected group and the control group. The results were considered statistically significant if *p* < 0.05, (*), *p* < 0.01 (**), or *p* < 0.001 (***).

## 3. Results

### 3.1. Effect of the 686 Infection on Live Cells in the Spleen

**Figure 1:**
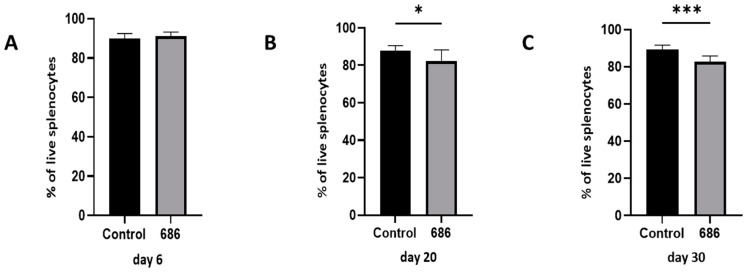
The effect of 686 infection on frequencies of live cell populations. After 686 infection, single cell suspensions from spleens of uninfected control and 686-infected groups were collected for flow cytometry analysis at three time points: 6 dpi (panel **A**), 20 dpi (panel **B**), and 30 dpi (panel **C**)). Cell viability dye, Live/Dead™ near-infrared (Invitrogen, Carlsbad, CA, USA), was used to exclude dead cells. Frequencies of live cells were analyzed at each time point. Using an unpaired *t*-test, each data point represents the mean percentage of cells from seven samples of the respective treatment, and the error bars represent the standard deviation. Asterisks above the group averages plotted in the graph indicate statistical significance between the groups; * *p* < 0.05, *** *p* < 0.001.

The live population of splenocytes was severely impacted by the 686 infection. This was manifested as a significant reduction in the percentage of live cells, beginning on day 20 and extending until 30 days post-infection (Figure 1B,C).

### 3.2. Effect of 686 Infection on T Cell Subsets in the Spleen (Figure 2, Figure 3 and Figure 4)

**Figure 2:**
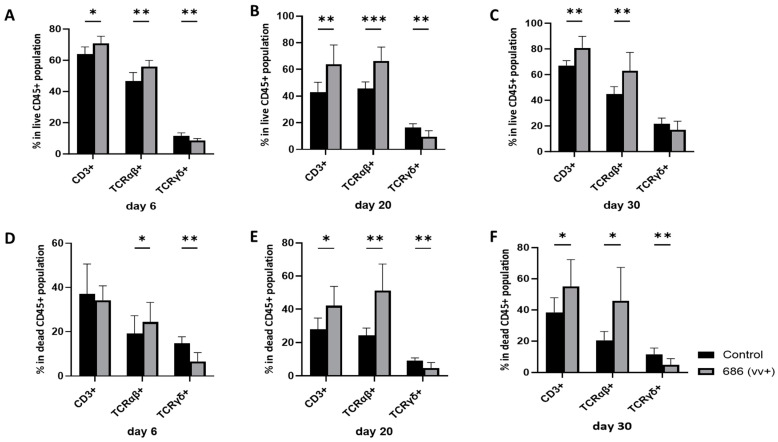
The effect of 686 infection on frequencies of T cell subsets within splenic CD45+ live and dead cell populations. After 686 infection, single cell suspensions from spleens of uninfected control and 686-infected groups were collected at three time points (6, 20, and 30 dpi) for flow cytometry analysis. Cell viability dye, Live/Dead™ near-infrared (Invitrogen, Carlsbad, CA, USA), was used to exclude dead cells. Frequencies of CD3+, TCRαβ+, and TCRγδ+ cells were measured within all CD45+ splenocytes in both live (**A**–**C**) and dead (**D**–**F**) populations. Using an unpaired *t*-test, each data point represents the mean percentage of cells from seven samples of the respective treatment, and the error bars represent the standard deviation. Asterisks above the group averages plotted in the graph indicate statistical significance between the groups; * *p* < 0.05, ** *p* < 0.01, *** *p* < 0.001.

The proportions of various T cell subsets in the spleen were assessed at 6, 20, and 30 days post-686 infection and compared with those in age-matched negative control chickens (results are summarized in Supplementary Table S2). In the 686-infected group, within the CD45+ cells, there was a significant increase in CD3+ and TCRαβ+ T cells in live cells on days 6, 20, and 30 (Figure 2A–C), and in dead cells at 20 and 30 dpi (Figure 2E,F). By contrast, the percentage of TCRγδ+ cells was significantly decreased on days 6 and 20 and numerically decreased on day 30 in live cells (Figure 2A–C), whereas it was significantly decreased at all time points in the dead cells (Figure 2D–F).

**Figure 3:**
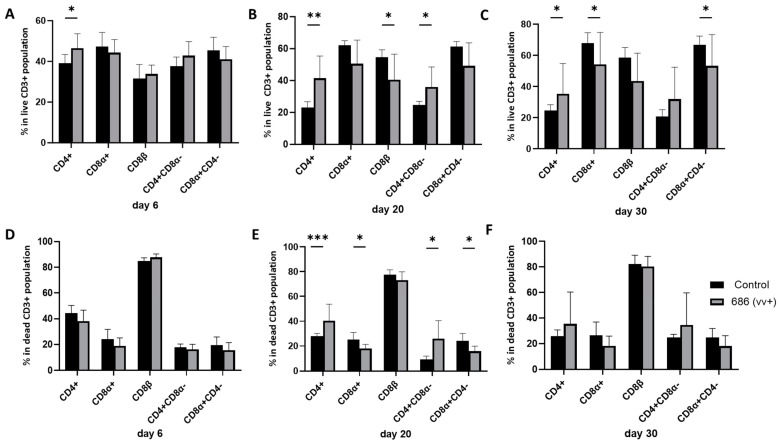
The effect of 686 infection on frequencies of T cell subsets within splenic CD3+ live and dead cell populations. After 686 infection, single cell suspensions from spleens of uninfected control and 686-infected groups were collected at three time points (6, 20, and 30 dpi) for flow cytometry analysis. Cell viability dye, Live/Dead™ near-infrared (Invitrogen, Carlsbad, CA, USA), was used to exclude dead cells. Frequencies of CD4+, CD8α+, CD8β+, CD4+CD8α-, and CD8α+CD4- T cells were measured within CD3+ splenocytes in both live (**A**–**C**) and dead (**D**–**F**) populations. Using an unpaired *t*-test, each data point represents the mean percentage of positive cells from seven samples of the respective treatment, and the error bars represent the standard deviation. Asterisks above the group averages plotted in the graph indicate statistical significance between the groups; * *p* < 0.05, ** *p* < 0.01, *** *p* < 0.001.

Further evaluation of CD3+ cells (results are summarized in Supplementary Table S3) revealed a statistically significant increase in the percentage of total CD4+ cells at all time points in live cells (Figure 3A–C) and at 20 days in dead cells (Figure 3E). A similar trend was observed when evaluating CD4+CD8- T cells, which increased significantly on day 20 (Figure 3B,E) and almost significantly (*p* = 0.09) on day 30 (Figure 3C,F) in both live and dead cell populations. Total CD8α+ T cells percentage showed a decline starting on day 20, reaching significance on day 30 in live cells (Figure 3B,C), and it was significantly decreased on day 20 in dead cells (Figure 3E). The percentage of CD4-CD8α+ T cells following the same trend that was observed in total CD8α+ T cells in both live and dead cell populations started to decrease at 20 dpi and significantly reduced by 30 dpi. (Figure 3). The percentage of CD8β+ T cells experienced a significant reduction on day 20 and a numerical decrease on day 30 in live cells (Figure 3B,E), but remained unaffected in dead cells at any time point (Figure 3).

**Figure 4:**
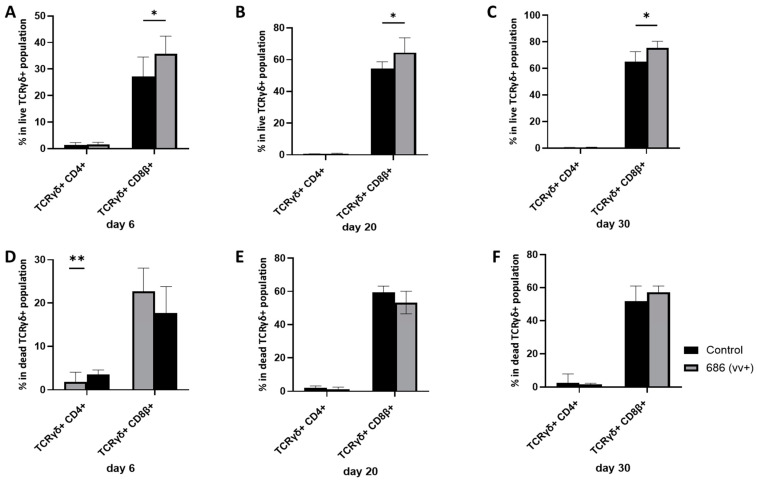
The effect of 686 infection on frequencies of different TCRγδ+ cell subsets within splenic live and dead cell populations. After 686 infection, single cell suspensions from spleens of uninfected control and 686-infected groups were collected at three time points (6, 20, and 30 dpi) for flow cytometry analysis. Cell viability dye, Live/Dead™ near-infrared (Invitrogen, Carlsbad, CA, USA), was used to exclude dead cells. Frequencies of TCRγδ+ CD4+ and TCRγδ+ CD8β+ cells were measured within TCRγδ+ splenocytes in both live (**A**–**C**) and dead (**D**–**F**) populations. Using an unpaired *t*-test, each data point represents the mean percentage of positive cells from seven samples of the respective treatment, and the error bars represent the standard deviation. Asterisks above the group averages plotted in the graph indicate statistical significance between the groups; * *p* < 0.05, ** *p* < 0.01.

A detailed analysis of TCRγδ cell subsets revealed a significant increase in the percentage of TCRγδ+CD8β+ cells at all three time points in live cells (Figure 4A–C), with a numerical decrease observed only on day 6 in the dead cell population (Figure 4D). Conversely, the percentage of TCRγδ+ CD4+ cells was unaffected at any time point in live cells but showed a significant increase only at 6 dpi in dead cells (Figure 4D).

### 3.3. Effect of 686 Infection on Expression of MHC-I and MHC-II in T Cells in Spleen

Expression levels of both MHC-I and MHC-II on T cells and T cell subsets were assessed by evaluating both frequency and median fluorescence intensity (MFI). MHC-I expression was examined on days 20 and 30 post-686 infection, while MHC-II was evaluated at 6, 20, and 30 dpi (results are summarized in Supplementary Table S4).

#### 3.3.1. MHC-I Expression (Table 1)

The percentage of CD3+MHC-I+ cell population was significantly increased on day 20 and day 30 in both live and dead cell populations. Concurrently, there was a significant increase in CD4+MHC-I+ cell proportion on days 20 and 30 in live cells and on day 20 in dead cells. However, the proportion of CD8β+ MHC-I+ cells was not affected at any time point in both live and dead cells (Table 1). The MFI analysis provided further confirmation for MHC-I expression levels on the same T cell subsets. MHC-I expression in CD3+ cells was significantly increased in live cells on days 20 and 30 and only on day 30 in dead cells. While MHC-I expression on CD4+ T cells showed a significant increase on day 30 in live cells and at both days (20 and 30) in dead cells. Even though the proportion of CD8β+MHC-I+ cells was not affected at any time point in both live and ad cells, MHC-I expression on CD8β+ T cells was increased significantly in both populations on day 30 (Table 1).

**Table 1:** Effects of vv+686 Infection on the Expression of Major Histocompatibility Complex type I (MHC-I) in the splenic T cells.

	Dpi ^3^	Treatment	CD3+ Cells (T Cells)	CD4+ T Cells		CD8β + T Cells		
			Live ^4^	Dead ^4^	Live ^4^	Dead ^4^	Live ^4^	Dead ^4^
**Expression of MHC-I ^1^ (Frequency)**	20	**Control**	23.11 ± 5.59 ^a^	49.24 ± 5.0 ^a^	6.066 ± 2.1 ^a^	3.790 ± 2.1 ^a^	22.39 ± 4.13 ^a^	59.41±5.90 ^a^
		**686**	35.43 ± 10.8 ^b^	62.91 ± 11.3^b^	19.06 ± 9.6 ^b^	6.531 ± 2.2 ^b^	18.90 ± 9.01 ^a^	48.87±12.4 ^a^
	30	**Control**	19.99 ± 2.87 ^a^	37.40 ± 10.8 ^a^	2.874 ± 0.7 ^a^	4.774 ± 3.3 ^a^	22.69 ± 5.68 ^a^	45.27±6.98 ^a^
		**686**	34.50 ± 9.89 ^b^	60.53 ± 10.6 ^b^	15.66 ± 14 ^b^	7.581 ± 5.0 ^a^	19.88 ± 9.96 ^a^	41.08 ± 17.8 ^a^
**Expression of MHC-I (MFI) ^2^**	20	**Control**	1840 ± 171.9 ^a^	3890 ± 895.2 ^a^	2568 ± 489.2 ^a^	2205 ± 519.2 ^a^	2091 ± 179.6 ^a^	4047 ± 945 ^a^
		**686**	2428 ± 408.1^b^	3729 ± 1062.6 ^a^	3212 ± 764.1 ^a^	3158 ± 904.3 ^b^	2282 ± 350.4 ^a^	3590 ± 1204 ^a^
	30	**Control**	1796 ± 119.4 ^a^	2812 ± 433.7 ^a^	2669 ± 144.6 a	1802 ± 127.3 ^a^	1918 ± 143.1 ^a^	2899 ± 414 ^a^
		**686**	2467 ± 528.2 ^b^	3542 ± 385.9 ^b^	3474 ± 361.9 ^b^	2971 ± 481.5 ^b^	2428 ± 267.1 ^b^	3422 ± 372 ^b^

^1^ MHC = Major histocompatibility molecule type I. ^2^ MFI = Median fluorescent intensity. ^3^ dpi = Days post-infection. ^4^ Results are presented as the average per group (7 samples per treatment group) ± standard deviation. Different superscript lowercase letters indicate that differences among groups were statistically significant in the unpaired *t*-test (*p* < 0.05).

#### 3.3.2. MHC-II Expression (Table 2)

The percentage of CD3+MHC-II+ in the live cells was increased numerically on days 20 and 30, but no difference was detected on day 6. In dead cells, CD3+ MHC-II+ T cell proportion was significantly higher on day 30, and no differences were seen on days 6 and 20. The percentage of CD4+ MHC-II+ T cells in live cells was significantly decreased on day 6, numerically increased at 20 dpi, and significantly increased at 30 dpi in the 686-infected group, but in dead cells, CD4+ MHC-II+ T cell percentage was significantly higher only on day 20. No difference was detected in the CD8β+ MHC-II+ T cell proportion at any time point in both live and dead cells (Table 2).

The MFI analysis revealed a significant increase of MHC-II expression on live CD3+ cells on days 20 and 30, but no difference was observed on day 6 post-686 infection. However, in dead CD3+ cells, a significantly lower MHC-II expression was detectable on days 6 and 20. The same trend for MHC-II expression on live CD3+ cells was observed in live CD4+ T cells; MHC-II was significantly increased on days 20 and 30, but there was no detectable difference on day 6 post-686 infection. In dead CD4+ T cells, MHC-II expression was significantly decreased on day 6. In live CD8β+ T cells, there was no difference at any time point, but in dead cells, MHC-II expression decreased significantly on CD8β+ T cells on days 6 and 20 post-infection (Table 2).

**Table 2:** Effects of vv+686 Infection on the Expression of Major Histocompatibility Complex Type II (MHC-II) in the Splenic T Cells.

	Dpi ^3^	Treatment	CD3+ Cells (T Cells)		CD4+ T Cells		CD8β + T Cells	
			Live ^4^	Dead ^4^	Live ^4^	Dead ^4^	Live ^4^	Dead ^4^
**Expression of MHC-II ^1^ (Frequency)**	6	**Control**	31.64 ± 6.83 ^a^	45.50 ± 6.15 ^a^	25.27 ± 4.5 ^a^	1.521 ± 0.6 ^a^	19.11 ± 7.26 ^a^	43.67 ± 3.38 ^a^
		**686**	26.74 ± 4.47 ^a^	42.53 ± 2.82 ^a^	19.57 ± 1.5^b^	1.530 ± 0.6 ^a^	15.78 ± 3.92 ^a^	47.24 ± 3.22 ^a^
	20	**Control**	23.79 ± 8.41 ^a^	47.89 ± 5.00 ^a^	13.90 ± 5.2 ^a^	2.627 ± 1.2 ^a^	25.71 ± 8.16 ^a^	58.97 ± 5.78 ^a^
		**686**	29.49 ± 5.28 ^a^	53.83 ± 6.6 ^a^	19.21 ± 7.4 ^a^	4.247 ± 1.6 ^b^	18.54 ± 8.68 ^a^	49.36 ± 12.5 ^a^
	30	**Control**	23.70 ± 7.87 ^a^	32.89 ± 10.9 ^a^	8.30 ± 3.4 ^a^*	3.807 ± 1.9 ^a^	23.04 ± 8.10 ^a^	51.73 ± 7.71 ^a^
		**686**	33.91 ± 11.5 ^a^	49.03 ± 6.61 ^b^	18.77 ± 15 ^b^*	4.896 ± 4.2 ^a^	20.04 ± 5.94 ^a^	49.78 ± 20.6 ^a^
**Expression of MHC-II (MFI) ^2^**	6	**Control**	2151 ± 271 ^a^	53,336 ± 16,034 ^a^	1890 ± 161 ^a^	12,978 ± 2102 ^a^	2460 ± 548 ^a^	53,361 ± 18,348 ^a^
		**686**	2195 ± 239 ^a^	22,765 ± 10,118 ^b^	1907 ± 233 ^a^	8661 ± 3291 ^b^	2400 ± 309 ^a^	19,952 ± 9572 ^b^
	20	**Control**	2716 ± 347 ^a^	22,501 ± 8058 ^a^	1804 ± 229 ^a^	4179 ± 1098 ^a^	1946 ± 240 ^a^	27,155 ± 11,082 ^a^
		**686**	3141 ± 337 ^b^	8024 ± 4465 ^b^	2322 ± 366 ^b^	4279 ± 1324 ^a^	2087 ± 287 ^a^	8179 ± 4432 ^b^
	30	**Control**	1684 ± 296 ^a^	21,109 ± 5955 ^a^	1572 ± 228 ^a^	6899 ± 1344 ^a^	1673 ± 297 ^a^	23,445 ± 8857 ^a^
		**686**	2750 ± 1102 ^b^	18,092 ± 1212 ^a^	2767 ± 1091^b^	7251 ± 855 ^a^	2214 ± 921 ^a^	19,933 ± 13,066 ^a^

^1^ MHC = Major histocompatibility molecules. ^2^ MFI = Median fluorescent intensity. ^3^ dpi = Days post-infection. ^4^ Results are presented as the average per group (7 samples per treatment group) ± standard deviation. Different superscript lowercase letters indicate that differences among groups were statistically significant in the unpaired *t*-test (*p* < 0.05. * Data was not normally distributed, so a nonparametric Mann-Whitney test was performed comparing median and 95% CI for detecting difference.

### 3.4. Effect of 686 Infection on B Cells and Macrophages in Spleens (Figure 5)

**Figure 5:**
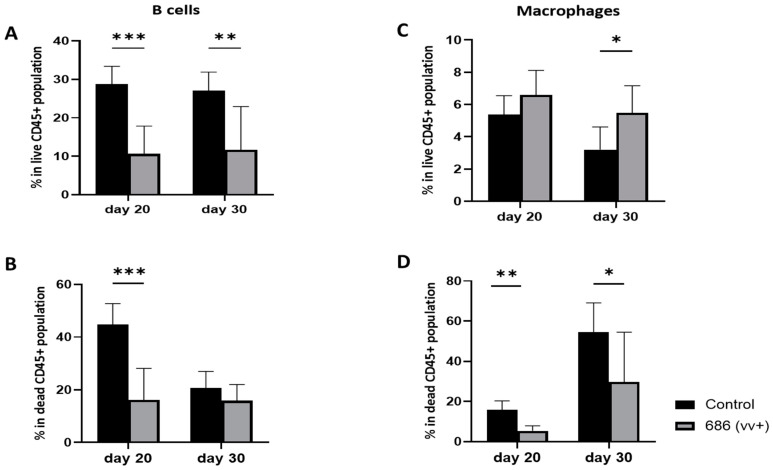
Effect of 686 infection on frequencies of B cells and macrophages within splenic CD45+ live and dead cell populations. After 686 infection, single cell suspensions from spleens of uninfected control and 686-infected groups were collected at two time points (20 and 30 dpi) for flow cytometry analysis. Cell viability dye, Live/Dead™ near-infrared (Invitrogen, Carlsbad, CA, USA), was used to exclude dead cells. Frequencies of B cells and macrophages were measured within CD45+ splenocytes in both live (**A**,**C**) and dead (**B**,**D**) populations. Using an unpaired *t*-test, each data point represents the mean percentage of positive cells from seven samples of the respective treatment, and the error bars represent the standard deviation. Asterisks above the group averages plotted in the graph indicate statistical significance between the groups; * *p* < 0.05, ** *p* < 0.01, *** *p* < 0.001.

The percentages of B cells and macrophages were examined at 20 and 30 days post-infection and compared to age-matched negative control chickens. The percentage of B cells in the 686-infected group were significantly reduced in live cells at both 20 and 30 dpi (Figure 5A). However, in dead cells, this significant decrease was detectable only on day 20 after the 686 infection (Figure 5B). In contrast, the percentage of macrophages was significantly increased at 30 days post-infection in the live cell population (Figure 5C). However, in the dead cell population, a significant decrease in macrophage percentages was seen at both 20 and 30 days following the 686 infection (Figure 5D).

## 4. Discussion

We have previously demonstrated that vv+MDV 686 strain significantly reduced the live population of splenocytes by 28–30 days in commercial meat-type chickens bearing maternal antibodies against MDV [25]. In the present study, our objective was to evaluate the effect of early infection with 686 strain on temporal changes in different splenic immunophenotypes, amongst both live and dead cell populations, on days 6, 20, and 30 dpi. Our results found a significant reduction in the total live cell population starting at 20 dpi, primarily affecting B cells, CD8β+, and γδ T cells, while a significant increase in the frequencies of both live and dead CD3+ and CD4+ T cells was observed at 20 dpi. Analysis of MHC expression showed that the CD3+ and CD4+ T cell expression of MHC-I was significantly higher at 20 and 30 dpi, whereas the MHC-II expression on these cells was significantly reduced at 6 dpi but was later found upregulated by 30 dpi. Collectively, it seems that maternal antibodies might delay the damage induced by the vv+MDV 686 strain in the splenic immune cells. However, protection is limited as the negative effects can be detected as early as 20 dpi. These findings are particularly relevant in broiler chicken flocks that are not vaccinated against MDV.

Previous studies demonstrate that maternal antibodies provide protection against early cytolytic infection and subsequent lymphoid organ atrophy even with highly virulent strains of MDV [16,17,20,21]. However, they do not confer any protection against infection, shedding of the virus, or development of tumors [22]. Furthermore, protection conferred by maternal antibodies can be compromised by high doses of virulent MDV strains [14,16,18,19] and weans away by 21 days [15,17,29]. In a previous study, we demonstrated that vv+, to a certain extent vv, but not vMDV, induce severe lysis of splenocytes in maternal antibody-positive chickens at 28–30 dpi [25]. The results of the present study confirm that such destruction of splenocytes can be observed at 20 dpi onward but not at 6 dpi, suggesting that the presence of maternal antibodies can delay the onset of the virus replication in lymphoid organs. Furthermore, we have characterized the affected cells as B cells, γδ T cells, CD8α T cells, and CD8β T cells. Because of the delay in the early cytolytic infection, there is an overlap between early cytolytic infection, reactivation from latency, and the development of tumors. This could explain why in our study, starting at 20 dpi, there was a severe reduction in live cells but a significant increase in CD4+ MHC-II+ cells, which might indicate a stage of lymphoproliferation.

B cells have been identified as the initial target for MDV infection [30], followed by activated T cells in which the virus could establish latency [9,30]. In maternal antibody-negative chickens, early cytolytic infection occurs between 4–6 days after infection. In the current study, we demonstrated that the percentage of B cells greatly decreased at 20 and 30 days of age, presumably attributable to the decline of maternal antibodies by this time. In previous studies conducted by Islam et al. (2002) using maternal antibody-positive broiler chickens, early cytolytic infection was not detected in the lymphoid organs until 22 days of age, which was accompanied by a significant decrease of B cells in the blood from 22 dpi onwards [22]. In that study, they could not detect a decrease in the percentage of B cells at 3, 7, 10, and 14 days after infection with vvMDV strain MPF 57. Because B cells were not evaluated at 6 days of age in our study, it is unknown whether B cells would have been affected at early time points when using the vv+MDV strain 686 for infection. Further studies are warranted to evaluate the effect of this strain on B cells at early ages in maternal antibody-postive commercial meat-type chickens.

In the context of T cell defense against MDV, CD8+ T cells, specifically cytotoxic T lymphocyte (CTL) responses, are critical. It has been reported that MDV infection delayed rejection of Rous sarcomas or MD transplantable tumor cells [31], blocked specific cytotoxic activities against MD tumor cells [32,33], and jeopardized protection conferred by ILT vaccines [7]. In the present study, we detected a decrease in the percentage of CD8α+ T cells in live cells at 20 and 30 dpi, with a clear and significant decrease in the percentage of CD8β+ T cells (CTLs) at 20 dpi but not on day 6. Reducing CTL cells could represent an immune evasion strategy for MDV; however, the mechanism by which it affects CTLs remains elusive. The decrease in the percentage of CD8α+ T cells and CD8β+ T cells observed in our study could be due to cell death or downregulation of CD8 molecules in these cells. Morimura et al. (1995), using commercial layer chickens challenged at 5 days of age with the vvMDV Md5 strain, demonstrated that CD8 α and β chains were downregulated at the transcriptional level in the periphery and spleen at 3 weeks after infection [34]. These authors also detected a downregulation of CD8 expression on both CD4-CD8+ and CD4+CD8+ thymocytes at one week after infection [35]. In the present study, it was not possible to determine whether the reduced percentage of CD8 is due to cell death or reduced transcription of CD8, and further studies are warranted. However, we did not detect an increase in the percentage of CD8+ T cells in the dead cell population at any timepoint, suggesting that the decreased percentage of CD8+ T cells may have been due to the downregulation of CD8 molecule [34,35].

Chickens are known to have a high abundance of γδ T cells, albeit their function is still poorly understood [36]. They can perform effector functions by producing a variety of cytokines and IFN-γ [37,38], exhibit cytotoxic activity [39], and play regulatory roles [40]. Previous studies have demonstrated that in chickens, the frequency of CD8+ γδ T cells increased following viral or bacterial infection in the spleen, blood, and mucosal tissues [41,42,43]. Additionally, there is emerging evidence regarding the involvement of chicken γδ T cells in the immunity against MDV [42,44]. CVI988 vaccination induced a significantly higher percentage of CD8α+γδ T cells in the spleen and lung at 3 and 7 dpi, suggesting their role in immune response [42]. In our study, we detected a decrease in the frequency of γδ T cells at all time points (6, 20, and 30 dpi), which comes in accordance with the findings of Laursen et al. (2018), who showed that vvMDV infection in maternal antibody-negative chickens (SPF) decreased the frequencies of γδ T cells at 4, 10, and 21 dpi, but they were increased at 21 dpi when absolute numbers were considered. They also showed that almost all the increased γδ T cells expressed CD8 molecules [38]. In the current study, even though the decrease in the frequency of total γδ+ T cells was pronounced at all time points, investigating the phenotypes within γδ T revealed a significant increase in CD8β+ γδ+ T cells. It is possible that such a decrease in the frequency of γδ T cells is a consequence to the increase in TCRαβ T cells. Further studies are needed to further elucidate the exact role and phenotype of γδ T cells in MDV pathogenesis and if CD8β+ γδ+ T cells play a determinant role in the immune response to MDV.

CD4+ T cells are well known as the target cells of MDV to establish latency leading to potential transformation [32,45,46,47,48]. Following MDV infection, at 21–28 dpi, latently infected lymphocytes migrate to various visceral organs and peripheral tissues, proliferate, and form lymphomas [49]. In the current study, we detected an expansion in TCRαβ from 6 dpi and onwards until 30 dpi, accompanied by a significant increase of CD4+ T cells. Previous research by Morimura et al. (1995) demonstrated that infection with the vvMDV Md5 strain transiently increased the proportion of TCR Vb1+ subset as early as 16 dpi in the peripheral blood and spleen, and they suggested that this increase was due to the expansion of Vβ1+ CD4+ T cells [34]. Later in 2008, Sarson et al. (2008) reported upregulation in the expression of the TCR-β chain gene at 14 dpi, which is the typical time point associated with the latency phase of MDV pathogenesis. They suggested that this increase might be due to expansion of CD4+ T cells and may be indicative of mechanisms that are associated with the initiation of T cell transformation and lymphoproliferation [50]. In our study, the observed expansion of TCRαβ and the increase of CD4+ T cells at 20 and 30 days can be explained by the development of lymphomas. However, the early expansion at 6 dpi was unexpected. It could be due to an inflammatory response mediated by the innate immune response cytokines in infected cells, such as IFN-γ, which induces T cell activation and differentiation [51]. However, further investigations are warranted.

Chicken macrophages are believed to play a potential role in both innate and adaptive immunity against MDV [33]. In addition to their ability to present MDV antigens in association with MHC class I and II molecules to initiate adaptive immunity, they can also be directly involved in the inhibition of MDV replication [33,52]. Macrophages exert their antiviral activities through production of nitrogen oxide (NO), which inhibits MDV replication in various tissues of infected chickens and is crucial for controlling viral replication in vivo [53,54,55]. Activated macrophages also have the ability to lyse MDV-derived tumor cells in vitro [56]. However, macrophages could also be involved in the development of the disease. They can be infected by highly virulent MDV, they are important for transferring MDV to other cells [57], and they are capable of jeopardizing the ability of lymphocytes to proliferate in vitro to various mitogens [58]. In addition, macrophages isolated from MDV-induced tumors function as tumor-associated macrophages (TAMS), which produce tumor-growth-promoting factors and suppress T cell responses, inducing immune suppression [59]. In our study, we detected a significant increase of the macrophage frequency at 30 dpi. Furthermore, we detected a significant decrease in the frequency of macrophages in the dead cell population, suggesting that the virus might be affecting their life span. It is possible that this increase in macrophages is involved in the immunosuppression elicited by vv+MDV. Further studies are warranted to determine the exact phenotype and function of the increased macrophage population detected in this stay at 30 dpi.

MHC-I downregulation is a common mechanism by which herpesviruses avoid immune responses (reviewed in [60]). Previous studies have demonstrated MDV’s ability to down-regulate MHC-I both in vitro [61,62,63,64] and in vivo [20,25]. In vitro, MDV down-regulated MHC-I in infected cells but upregulated MHC-I in non-infected surrounding cells [61]. In our study, we detected an increase in MHC-I expression on the T lymphocytes at 20 and 30 dpi in both live and dead cells. We did not evaluate infection in these cell populations, so it was not possible to determine if cells expressing higher levels of MHC-I were infected or not. Upregulation of MHC-I in lymphocytes was reported by Dalgaard et al. (2009) at 4 and 8 weeks following infection with vvMDV strain RB1B. In their study, upregulation of MHC-I seemed to be influenced by chicken genetics [65]. There are two types of MHC-I molecules (class I major, or BF-2, and minor, or BF-1) with different functions [64]. MDV prevented the downregulation of MHC molecule BF1, which specifically interacts with NK cells, thereby inhibiting NK-cell activation. On the other hand, MDV downregulated MHC- Ⅰ BF2, that binds to TCR on CD8+ T cells [65]. In our study, the monoclonal antibody used does not differentiate between BF1 and BF2, and therefore, it is unknown if the increased expression of MHC-I observed could be BF1 as reported by Kim et al. (2018). Furthermore, the monoclonal antibody used in this study cannot differentiate between intact MHC-I with peptide epitope and MHC-I with denatured peptide epitope (Hunt, personal communication). Further studies are warranted.

The effect of MDV infection on the expression of MHC-II seems to vary depending on the experimental model. Earlier studies on MDV pathogenesis demonstrated that T cells were only susceptible to MDV infection when expressing MHC-II [66]. Upregulation of MHC-II expression in cells infected with vvMDV strain Md11 both in vitro and in vivo has been reported [67]. However, expression of MHC-II in T cells varies at different points after infection. Dalgaard et al. (2009) reported that MDV GA strain infection in chickens carrying the B19 and B21 MHC haplotypes downregulates MHC class II expression on T cells at 1 week post-infection but upregulates it on different subsets of T cells by 4 weeks post-infection [65]. Yu et al. reported that after infection with MDV, MHC-II gene expression was up-regulated at 4 dpi, down-regulated at 14 and 28 dpi, and up-regulated again at 35 dpi in peripheral blood lymphocytes [68]. Also, some studies have shown downregulation of MHC-II expression in the spleen of MDV-infected chickens at all evaluation time points [50,69,70,71]. In our study, we have detected downregulation of MHC-II expression on T cell subsets at 6 dpi and upregulation at 20 and 30 dpi, similar to the results reported by Yu et al. [68] and Dalgaard et al. [65]. Our results suggest that MDV regulates MHC-II expression in different manners at different times of infection. Because early cytolytic infection was delayed due to the maternal antibodies, the virus downregulated MHC-II expression to avoid immune responses. Later in the pathogenesis, as the virus started replicating and needed more target cells, it upregulated MHC-II expression.

In conclusion, our study contributes to a deeper understanding of the interplay between vv+MDV and the host immune system in commercial meat-type chickens bearing maternal antibodies, which is essential for the development of effective strategies for disease control, including MDV-IS. We confirmed that there was a severe reduction in various immune cells (B cells, γδT cells, CD8α+, and CD8β+ T cells) at 20 and 30 dpi but not at 6 dpi. These findings suggest that maternal antibodies delayed replication of the virus but conferred little to no protection against the damage that MDV can cause to the spleen. Therefore, unvaccinated commercial meat-type chickens might suffer severe MDV-IS if exposed to a vv+MDV strain under field conditions. In a previous work, we demonstrated that vaccination with CVI988 plus HVT conferred protection against such damage [25]. However, it is unknown if other vaccination programs, including HVT or partial doses of HVT, will fully protect against the damage that vv+MDV strains can cause to the spleen at 20 and 30 dpi. Furthermore, our study opens new questions about the role of γδ+ CD8β+ T cells and macrophages in the pathogenesis of vv+MDV strains and emphasizes the need for further studies on the ability of MDV to regulate MHC-I and MHC-II in chickens.

## Data Availability

Research data are available upon request from Isabel M. Gimeno.

## Supplementary Materials

The following supporting information can be downloaded at: https://www.mdpi.com/article/10.3390/v16071092/s1. Figure S1: Supplementary: Gating strategy used in T cell phenoptyping; Panel A: The gating strategy used for the analysis included gating lymphocyte single-cell splenocytes using forward (FSC) and side (SSC) scatter parameters of width (W) and height (H) to exclude doublet cells, followed by gating on the live and dead cell populations. Panel B: The live and dead cell gates were drilled down to obtain CD3+ cells, which were further drilled to CD4+CD8-, CD4+CD8+, CD8+CD4-, and CD4-CD8- T cell subsets. Figure S2: Supplementary: Gating strategy used in different T cell subset phenotyping; Panel A: The gating strategy used for the analysis included gating lymphocyte single-cell splenocytes using forward (FSC) and side (SSC) scatter parameters of width (W) and height (H) to exclude doublet cells, followed by gating on the live and dead cell populations, then from live and dead cells a CD45+ gate was drilled. Panel B: Live and dead CD45+ cell gates were drilled down to obtain CD3+TCRγδ+ and CD3+TCRαβ+, and each gate of them was further drilled down to CD4+ cells and CD8β+ cells. Panel C: Live and dead CD45+ cell gates were drilled down to obtain CD3+ cells, which was further drilled to CD8β+ T cells. Figure S3: Supplementary: Gating strategy used for B cells and macrophages; Panel A: The gating strategy used for the analysis included gating lymphocyte single-cell splenocytes using forward (FSC) and side (SSC) scatter parameters of width (W) and height (H) to exclude doublet cells, followed by gating on the live and dead cell populations. Panel B: The live and dead cell gates were drilled down to obtain CD45+ Bu-1+ cells and CD45+ Kulo-1+ to detect B cells and macrophages subsequently. Figure S4: Supplementary: Gating strategy used for MHC-II expression on different T cell subsets; Panel A: The gating strategy used for the analysis included gating lymphocyte single-cell splenocytes using forward (FSC) and side (SSC) scatter parameters of width (W) and height (H) to exclude doublet cells, followed by gating on the live and dead cell populations. Panel B: The live and dead cell gates were drilled down to obtain CD3+ MHC-II+ cells, which were further drilled to CD4+MHC-II+ and CD8b+MHC-II+. Figure S5: Supplementary: Gating strategy used for MHC-I expression on different T cell subset phenotyping; Panel A: The gating strategy used for the analysis included gating lymphocyte single-cell splenocytes using forward (FSC) and side (SSC) scatter parameters of width (W) and height (H) to exclude doublet cells, followed by gating on the live and dead cell populations. Panel B: The live and dead cell gates were drilled down to obtain CD3+ MHC-I+ cells, which were further drilled to CD4+MHC-I+ and CD8β+MHC-I+. Table S1: Staining panels for flow cytometry analysis of days 6, 20, and 30 of age in meat-type chickens. Table S2: Summary table showing differences in the splenocyte populations between 686-infected meat-type chickens and negative controls at different time points post-infection in both live and dead cells. Table S3: Summary table showing differences in the spleen T cell subsets present (as a percentage of CD3 T cells) between 686-infected meat type chickens and negative controls at different time points post-infection in both live and dead cells. Table S4: Summary table showing major histocompatibility complex (MHC) class I and II expression on different T cell subsets of 686-infected chickens when compared to the uninfected controls at different time points post-infection in both live and dead cells.
