# Supplementary material for: Temporal Changes in Splenic Immune Cell Populations following Infection with a Very Virulent plus MDV in Commercial Meat-Type Chickens
Source: Viruses. 2024 Jul 6;16(7):1092. doi: 10.3390/v16071092 (PMC11281429; doi:10.3390/v16071092)
Supplement: Supplementary file 1 [file viruses-16-01092-s001.zip › Figure supplementary 1.pptx]

## Slide 1
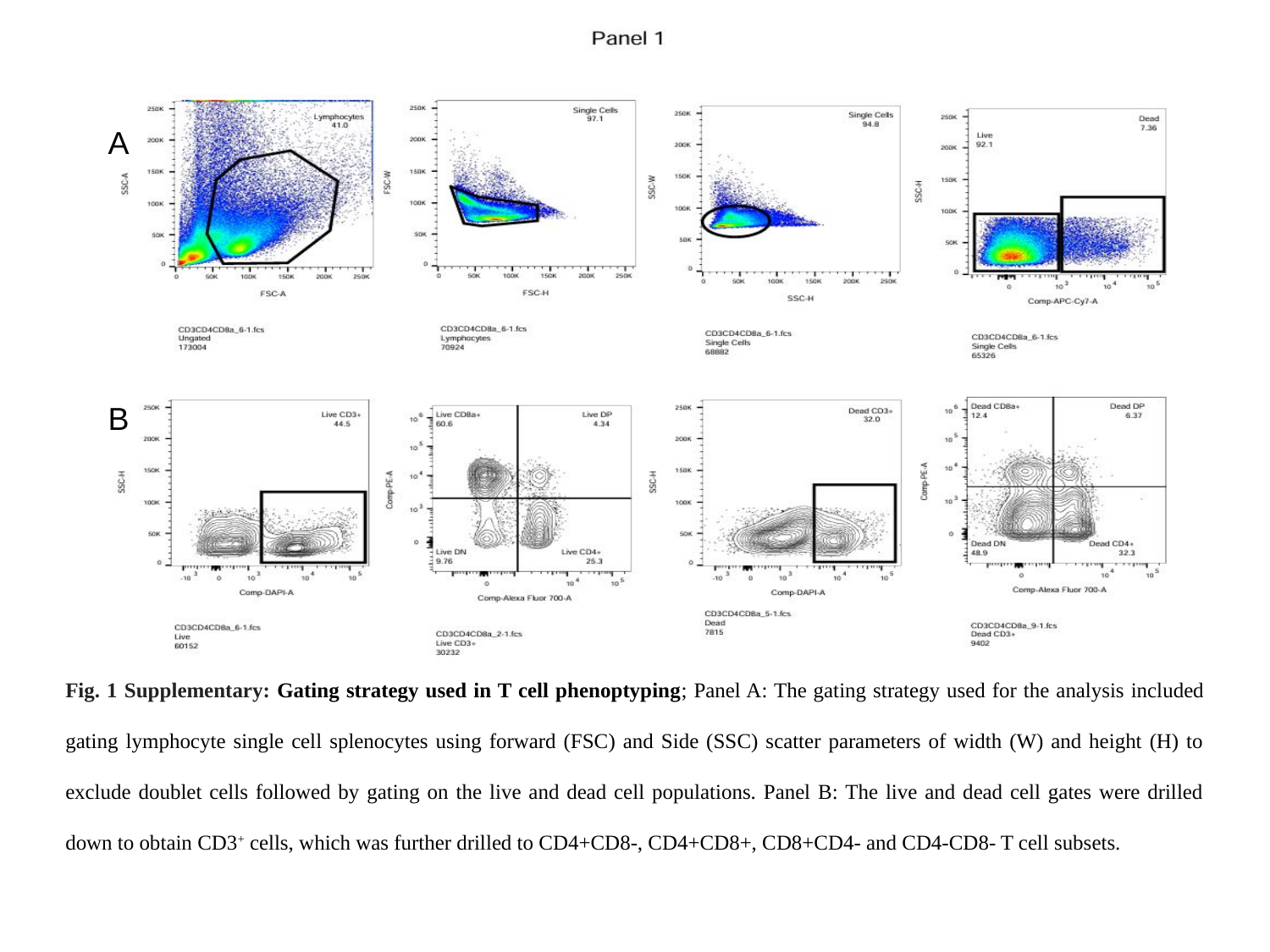

A
B
Fig. 1 Supplementary: Gating strategy used in T cell phenoptyping; Panel A: The gating strategy used for the analysis included gating lymphocyte single cell splenocytes using forward (FSC) and Side (SSC) scatter parameters of width (W) and height (H) to exclude doublet cells followed by gating on the live and dead cell populations. Panel B: The live and dead cell gates were drilled down to obtain CD3+ cells, which was further drilled to CD4+CD8-, CD4+CD8+, CD8+CD4- and CD4-CD8- T cell subsets.
