# Supplementary material for: Temporal Changes in Splenic Immune Cell Populations following Infection with a Very Virulent plus MDV in Commercial Meat-Type Chickens
Source: Viruses. 2024 Jul 6;16(7):1092. doi: 10.3390/v16071092 (PMC11281429; doi:10.3390/v16071092)
Supplement: Supplementary file 1 [file viruses-16-01092-s001.zip › Figure supplementary 2.pptx]

## Slide 1
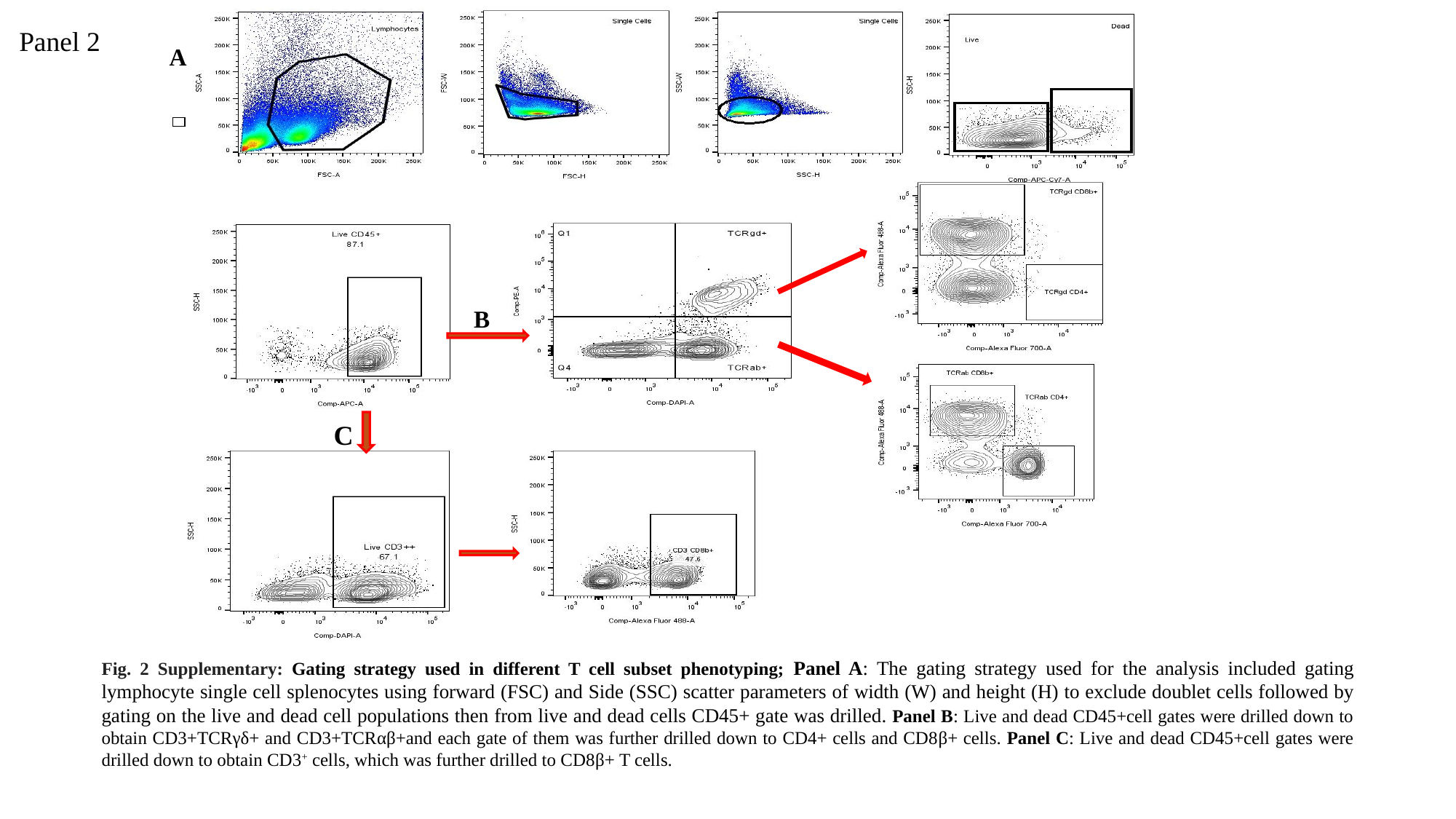

A
B
C
Panel 2
Fig. 2 Supplementary: Gating strategy used in different T cell subset phenotyping; Panel A: The gating strategy used for the analysis included gating lymphocyte single cell splenocytes using forward (FSC) and Side (SSC) scatter parameters of width (W) and height (H) to exclude doublet cells followed by gating on the live and dead cell populations then from live and dead cells CD45+ gate was drilled. Panel B: Live and dead CD45+cell gates were drilled down to obtain CD3+TCRγδ+ and CD3+TCRαβ+and each gate of them was further drilled down to CD4+ cells and CD8β+ cells. Panel C: Live and dead CD45+cell gates were drilled down to obtain CD3+ cells, which was further drilled to CD8β+ T cells.
