# Supplementary material for: Temporal Changes in Splenic Immune Cell Populations following Infection with a Very Virulent plus MDV in Commercial Meat-Type Chickens
Source: Viruses. 2024 Jul 6;16(7):1092. doi: 10.3390/v16071092 (PMC11281429; doi:10.3390/v16071092)
Supplement: Supplementary file 1 [file viruses-16-01092-s001.zip › Supplementary Table 2.pdf]

Supplementary Table 2: Summary table showing differences in the splenocytes populations between 686 infected meat type chickens and negative controls at different time points post infection in both live and dead cells.

Days post challenge	Total viable cells[1]	Frequency of B cells and Macrophage (from all CD45)[1]		Frequency of T cell subsets (from all CD45+)[1]		
		B cells	Macrophage	CD3+	TCRαβ	TCRγδ
6		NA[2]	NA[2]		*	↓
20		↓	↓	*	*	↓
30	*		↓	*	*	↓

[1] Data from 686 group is compared to the negative control group. Analysis of live cells is indicated by colors being grey (no statistically significant differences between 686 and negative control groups), blue (686 group had a significant increase of that cell population when compared to negative control group), and red (686 group had a significant decrease of that cell population when compared to negative control group). Analysis of dead cells is indicated by symbols. Lack of differences between group (no symbol), significant increase (*), and significant decrease (↓)

[2] NA= data was not available due to lack of availability of monoclonal antibodies
