# Supplementary material for: Temporal Changes in Splenic Immune Cell Populations following Infection with a Very Virulent plus MDV in Commercial Meat-Type Chickens
Source: Viruses. 2024 Jul 6;16(7):1092. doi: 10.3390/v16071092 (PMC11281429; doi:10.3390/v16071092)
Supplement: Supplementary file 1 [file viruses-16-01092-s001.zip › Supplementary Table 3.pdf]

Supplementary Table 3: Summary table showing differences in the spleen T cell subsets present (as a percentage of CD3 T cells) between 686 infected meat type chickens and negative controls at different time points post infection in both live and dead cells.

Days post challenge	Frequency of T cell subsets (from all CD3+)[1]					Frequency of TCRγδ subsets[1]	
	CD4+	CD8α+	CD8β+	CD4+CD8α-	CD8α+CD4-	CD4+	CD8β+
6						*	
20	*	↓		*	↓		
30							

[1] Data from 686 group is compared to the negative control group. Analysis of live cells is indicated by colors being grey (no statistically significant differences between 686 and negative control groups), dark blue (686 group had a significant increase of that cell population when compared to negative control group), and dark red (686 group had a significant decrease of that cell population when compared to negative control group). Light shadows of blue and red indicate that differences were numerical and approaching significance. Analysis of dead cells is indicated by symbols. Lack of differences between group (no symbol), significant increase (*), and significant decrease (↓)
