# Supplementary material for: Temporal Changes in Splenic Immune Cell Populations following Infection with a Very Virulent plus MDV in Commercial Meat-Type Chickens
Source: Viruses. 2024 Jul 6;16(7):1092. doi: 10.3390/v16071092 (PMC11281429; doi:10.3390/v16071092)
Supplement: Supplementary file 1 [file viruses-16-01092-s001.zip › Supplementary Table 4.pdf]

Supplementary Table 4: Summary table showing Major histocompatibility complex (MHC) class I and II expression on different T cell subsets of 686 infected chickens when compared to the uninfected controls at different time points post infection in both live and dead cells.

**MHC-I Expression on T cells[1]**							
Days post challenge	Unit	CD3+		CD4+T cells		CD8β+ T cells	
		live	dead	live	dead	live	dead
20	%						
	MFI[2]						
30	%						
	MFI						
**MHC-II Expression on T cells[1]**							
Days post challenge	Unit	CD3+		CD4+T cells		CD8β+ T cells	
		live	dead	live	dead	live	dead
6	%						
	MFI						
20	%						
	MFI						
30	%						
	MFI						

[1] Data from 686 group is compared to the negative control group. Analysis of live cells is indicated by colors being grey (no statistically significant differences between 686 and negative control groups), dark blue (686 group had a significant increase of that cell population when compared to negative control group), and dark red (686 group had a significant decrease of that cell population when compared to negative control group). Light shadows of blue and red indicate that differences were numerical and approaching significance.

[2] Median fluorescent intensity
