# Supplementary material for: Temporal Changes in Splenic Immune Cell Populations following Infection with a Very Virulent plus MDV in Commercial Meat-Type Chickens
Source: Viruses. 2024 Jul 6;16(7):1092. doi: 10.3390/v16071092 (PMC11281429; doi:10.3390/v16071092)
Supplement: Supplementary file 1 [file viruses-16-01092-s001.zip › Supplementary Table 1.pdf]

Supplementary table 1: Staining panels for flow cytometry analysis of day 6*, 20, and 30 of age in meat type chickens

**Panel**[1]	**Antigen**	**Clone**	**Fluorochrome**
1			
	-	-	Live/Dead (L/D) Near Infra-red (IR)[2]
	CD3	CT-3	Pacific Blue (PB)™
	CD8α	CT-8	R-phycoerythrin (PE)
	CD4	CT-4	Alexa Fluor® 700 (AF®700)
2			
	-	-	Live/Dead (L/D) Near Infra-red (IR)
	CD45	LT40	Allophycocyanin (APC)
	CD3	CT-3	Pacific Blue (PB)™
	CD8β	EP42	Fluorescein Isothiocyanate (FITC)
	CD4	CT-4	Alexa Fluor® 700 (AF®700)
	TCR-γ/δ	TCR 1	R-phycoerythrin (PE)
3			
	-	-	Live/Dead (L/D) Near Infra-red (IR)
	CD45	LT40	Allophycocyanin (APC)
	CD3	CT-3	Pacific Blue (PB)™
	Monocyte/macrophage	KUL01	Alexa Fluor® 488 (AF®488)
	CD4	CT-4	Alexa Fluor® 700 (AF®700)
	Bu-1	AV20	R-phycoerythrin (PE)
4			
		-	Live/Dead (L/D) Near Infra-red (IR)
	CD45	LT40	Allophycocyanin (APC)
	CD3	CT-3	Pacific Blue (PB)™
	CD8β	EP42	Fluorescein Isothiocyanate (FITC)
	CD4	CT-4	Alexa Fluor® 700 (AF®700)
	MHC-II	CIa	R-phycoerythrin (PE)
5			
	-	-	Live/Dead (L/D) Near Infra-red (IR)
	CD45	LT40	Allophycocyanin (APC)
	CD3	CT-3	Pacific Blue (PB)™
	CD8β	EP42	Fluorescein Isothiocyanate (FITC)
	CD4	CT-4	Alexa Fluor® 700 (AF®700)
	MHC-I	F21-2	R-phycoerythrin (PE)

[1] Five panels were used in this study: Panel 1 and panel 2 used antibodies directed against different T cell subsets including chicken T cell receptor (TCR) gamma delta (γδ) cells ; Panel 3 used antibodies directed against chicken monocyte/macrophages (KULO1^{+}) and B cell lymphocytes (Bu-1); Panel 4 used antibodies directed to detect MHC-II surface expression on different T cell subsets; Panel 5 used antibodies directed to detect MHC-II surface expression on different T cell subsets.

[2] In all panels, exclusion of dead cells was performed by staining the cells with the Live/Dead™ Fixable Near-Infra-red (IR) Dead Cell Stain (Invitrogen, Carlsbad, CA).

*On day 6 only panel l, 2, and 4 were used due to unavailability of antibodies.
